# Spontaneous Scaling of a Primary Care Innovation in Real-Life Conditions: Protocol for a Case Study

**DOI:** 10.2196/54855

**Published:** 2023-12-28

**Authors:** France Légaré, Diogo G V Mochcovitch, Roberta de Carvalho Corôa, Amédé Gogovor, Ali Ben Charif, Cynthia Cameron, Annie Plamondon, Marie Cimon, Sabrina Guay-Bélanger, Geneviève Roch, Maxine Dumas Pilon, Jean-Sébastien Paquette, Robert K D McLean, Andrew Milat

**Affiliations:** 1 Department of Family Medicine and Emergency Medicine Faculty of Medicine Université Laval Quebec, QC Canada; 2 VITAM - Centre de Recherche en Santé Durable Centre Intégré Universitaire de Santé et de Services Sociaux de la Capitale-Nationale Quebec, QC Canada; 3 Centre de Recherche du CHU de Québec - Université Laval CHU de Québec Quebec, QC Canada; 4 CubecXpert Quebec, QC Canada; 5 Groupe de Médecine de Famille Universitaire de Lévis Centre Intégré de Santé et de Services Sociaux de Chaudière-Appalaches Lévis, QC Canada; 6 Faculty of Social Sciences Université Laval Quebec, QC Canada; 7 Faculty of Nursing Université Laval Quebec, QC Canada; 8 Indigo Clinic Montreal, QC Canada; 9 Department of Family Medicine McGill University Montreal, QC Canada; 10 Quebec College of Family Physicians Montreal, QC Canada; 11 International Development Research Centre Ottawa, ON Canada; 12 School of Public Health University of Sydney Camperdown, New South Wales Australia

**Keywords:** scaling, spread, primary care, spontaneous, knowledge translation, implementation science, scaling science

## Abstract

**Background:**

Scaling effective primary care innovations to benefit more people is of interest to decision makers. However, we know little about how promising innovations are being scaled “spontaneously,” that is, without deliberate guidance.

**Objective:**

We aim to observe, document, and analyze how, in real-life conditions, 1 primary care innovation spontaneously scales up across Quebec, Canada.

**Methods:**

We will conduct a participative study using a descriptive single-case study. It will be guided by the McLean and Gargani principles for scaling and reported according to the COREQ (Consolidated Criteria for Reporting Qualitative Research) guidelines. Informed by an integrated knowledge translation approach, our steering committee will include patient users throughout the project. Inspired by the Quebec College of Family Physician’s “Dragons’ Den” primary care program, we will identify a promising primary care innovation that is being or will be scaled spontaneously. We will interview the innovation team about their scaling experiences every month for 1 year. We will conduct interviews and focus groups with decision makers, health care providers, and end users in the innovation team and the target site about their experience of both scaling and receiving the scaled innovation and document meetings as nonparticipant observers. Interview transcripts and documentary data will be analyzed to (1) compare the spontaneous scaling plan and implementation with the McLean and Gargani principles for scaling and (2) determine how it was consistent with or diverged from the 4 McLean and Gargani guiding principles: justification, optimal scale, coordination, and dynamic evaluation.

**Results:**

This study was funded in March 2020 by the Canadian Institutes of Health Research. Recruitment began in November 2023 and data collection began in December 2023. Results are expected to be published in the first quarter of 2024.

**Conclusions:**

Our study will advance the science of scaling by providing practical evidence–based material about scaling health and social care innovations in real-world settings using the 4 guiding principles of McLean and Gargani.

**International Registered Report Identifier (IRRID):**

PRR1-10.2196/54855

## Introduction

The subject of scaling has long been present in various knowledge areas such as renewable energy sources [1], sustainability [2], and education [3]. Scaling is now rapidly gaining the attention of decision makers in health and social care, particularly in high-income countries [4]. In the literature and practice record, there are numerous means proposed for scaling innovations [5]. Many of these propose modifiers, such as scaling up or out and vertical or horizontal scaling, are used in an attempt to describe the different ways in which innovations are scaled or spread. ExpandNet, a World Health Organization–affiliated group, has produced several widely used practical guides on the topic and defines scaling up as an action that aims to increase the impacts of successfully tested innovations to benefit more people [6]. Thus, it is no surprise that alongside this World Health Organization practice–oriented initiative, scaling science is emerging as a distinct complement to applied or implementation science [7-9]. The term “scaling science” has a double meaning: the first referring to approaches to bringing innovations to optimal scale for the public good and the second referring to the critical and systematic (ie, scientific) study of these approaches to scaling [7,8].

Unfortunately, Canada is a country notorious for promising pilot projects that do not get scaled [10]. Explanations for this include the fact that Canada is comprised of multiple distinct health and social care systems [11]. In addition, the most effective scaling seems to occur in low- and middle-income countries, which may reduce the opportunities for decision makers from high-income countries to improve their knowledge and competencies in scaling [5]. Another potential explanation lies in the complexity of scaling health and social innovations [12,13] and the methodological traditions that prioritize testing with randomized controlled trials before scaling up [14,15]. This contributes to lengthening the scaling process, while decision makers are often focused on short-term impact. Finally, there is increasing pressure on high-income countries to reduce waste and implement effective innovations already scaled in low- and middle-income countries [16]. Now that high-income countries face a rapidly changing society with increasing resource constraints, effective scaling of beneficial innovations has become a high priority [17].

Most scaling occurs in the absence of scaling guidelines [5]. While there are attempts to establish rigorous and standardized guidelines, there is also a pressing need for studies that look at how scaling currently occurs on the ground when a promising or effective health care innovation is naturally or “spontaneously” expanded to reach a broader population. We adopted ExpandNet’s definition of spontaneous scaling as “diffusion of the innovation without deliberate guidance” [18]. The likelihood of spontaneous scaling is highest when the innovation effectively meets a clearly recognized need in a population or when a significant and urgent harmful event brings attention to the need to scale, for example, the COVID-19 pandemic [18]. Although the effectiveness of the innovation to be scaled should be demonstrated by its positive impacts on the public good, that is, it improves the well-being of individuals and populations through an increase in the quality of care [5,8], many experts and decision makers recognize that this is not always possible when facing a public health emergency [15,19]. Observation and analysis of spontaneous scaling are necessary to capture this process of adjusting plans and practice in real-life situations and will inform the knowledge base of scaling. Therefore, we aimed to observe, document, and analyze the spontaneous scaling (ie, that reflects the needs of a population but does not follow guidelines) of a promising primary care innovation within a specific health care context, compare it with the guiding principles of scaling described by McLean and Gargani [7-9], and make recommendations.

## Methods

### Overview of Study Design and Context

This study is a descriptive case study [20-22] adhering to the Rosenberg and Yate [23] recommendations for case study designs, which aim to comprehensively explore a particular case, including exploration of the phenomenon of interest and its contextual factors. Specifically, we define the case as the spontaneous process of scaling a promising primary care innovation within a specific health care context. The health care context is 2 large regional primary care organizations (an Integrated Health and Social Services Center [*Centre Intégré de Santé et de Services Sociaux*; CISSS] and an Integrated University Health and Social Services Center [*Centre Intégré Universitaire de Santé et de Services Sociaux*; CIUSSS]) within the Quebec health and social care services system. These 2 organizations are the administrative entities responsible for the delivery of health and social care services within their designated regions and for coordinating and integrating various facilities, such as hospitals, long-term care centers, rehabilitation centers, and community health centers.

### Case Study Approach

To gain insights into the real-life spontaneous scaling process of a promising innovation, we will delve into a specific case and draw broader conclusions about the phenomenon at conclusions. This research will also take an interpretative epistemological approach [24] to understand the context of the case from different perspectives. By using these approaches, we will shed light on the various factors and challenges associated with the scaling of spontaneous primary care innovations in real-world settings. We will report our findings using the COREQ (Consolidated Criteria for Reporting Qualitative Research) checklist [25].

### Scaling Framework

The documentation and analysis of the scaling process for this case study will be guided by a framework inspired by guiding principles proposed by McLean and Gargani [7-9]. This framework is based on a retrospective review of over 200 research and innovation projects funded by Canada’s International Development Research Centre. The choice to use the McLean and Gargani principles was influenced by three factors: (1) they allow for adaptability and flexibility throughout all phases of the scaling process, which is coherent with a spontaneous approach and with the real-life context of this study (ie, unexpected changes can be accommodated and plans adapted); (2) the guidelines apply an equity lens and emphasize the importance of ethics in assessing scaling impacts; and (3) the principles support our aim of focusing on the impacts of scaling on the public good, a focus that promotes placing the main emphasis on the end users in all scaling initiatives.

The 4 guiding principles for scaling impact described by McLean and Gargani [8,9] are justification, optimal scale, coordination, and dynamic evaluation. “Justification” addresses both ethical and technical issues. Before asking how to scale, one must ask why or whether to scale and about its technical feasibility. For example, does this web-based course for social workers have the necessary infrastructure to reach more professionals (technical justification)? What kind of impact on the public good will the course provide (ethical justification)? “Optimal scale” challenges the logic of “the more the merrier” and involves making informed decisions about choosing a scale that creates an optimal mix of magnitude, variety, sustainability, and equity [7]. This requires careful consideration of the potential benefits and risks and requires judgment in balancing desirable and undesirable impacts. For instance, if the course is scaled up to 1000 social workers but is only available temporarily, and only to those working in private settings, scale-up meets the magnitude goals but not the sustainability or equity goals. “Coordination” addresses relational issues; demands flexible articulation among the complex systems; and connects the diverse actors in the scaling process, such as the initiators, the enablers, the competitors, and the people affected by scaling. For example, coordination among those involved in identifying the need for the social workers’ course, those who developed the course, those who adapted and scaled it to further contexts, those who are evaluating it, those who are receiving the scaled-up version, and those who are receiving services from the newly trained social workers. For example, coordination in the case of the social workers' course would involve coordination among those who identified the need for the course, the course developers, those responsible for adapting and scaling it to diverse contexts, the evaluators assessing its effectiveness, and recipients of the scaled version. Coordination involves complexity, and this involves flexibility that requires an iterative process [8]. “Dynamic evaluation” addresses assessments in all phases of scaling: before, during, and after. Dynamic evaluation is not a linear before-after comparative assessment, but a constant application of evaluative thinking to check and adapt when necessary since scaling is a process that can change depending on the context of the site and innovation.

### Coproduction Approach and Project Governance

Following the integrated knowledge translation approach [26,27], this study embraces the active engagement of knowledge users as equal partners working alongside researchers in all research steps. Through coproduction [28,29], we aim to generate knowledge that is valuable and meaningful to all stakeholders involved in the research process.

To ensure effective project governance, the research team will establish a steering committee consisting of key stakeholders who will provide expert advice, recommendations, and feedback on questions as they arise. This committee will oversee and coordinate each stage of the study and focus on topics that directly impact the project. The committee members will include the project leader; study coordinator; coinvestigators (1 or 2); knowledge users (1 or 2); and end users, who may include citizen or patient partners (1 or 2). This diversity of stakeholders ensures inclusive representation throughout the project’s duration.

### Participants

#### Criteria for Choice of Innovation, Innovation Team, and Target Site

We will choose an innovation from a previous edition of the *Innovation Symposium*, a program organized by the Quebec College of Family Physicians inspired by the TV program “The Dragon’s Den” [30]. This event allows innovators, most of whom are from a clinical context and not from a research background, to pitch their promising innovations to clinical leaders and influential stakeholders (the Dragons) in Canada’s primary care ecosystem. If the Dragons consider the innovation promising, they will contribute toward accelerating its growth and scaling it. Based on our research team’s firsthand experience as attendees at previous symposiums (2017, 2019, and 2022), we will identify a spontaneous primary care innovation with considerable potential for scaling.

**Figure 1:**
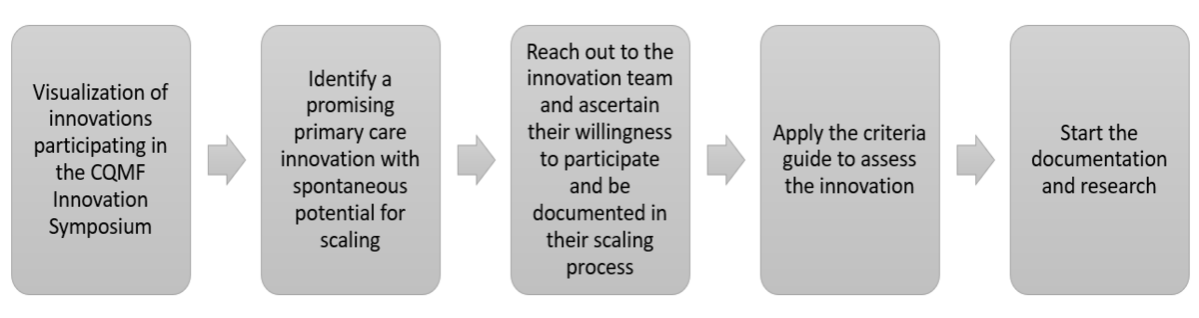
Process flow for choosing a promising innovation. CQMF: Quebec College of Family Physicians.

We will determine if the team is willing to participate in the project and, through a three-part questionnaire, whether (1) the innovation, (2) the innovation team, and (3) the context in which the innovation is embedded meet the following inclusion criteria based on our previous work [31-33] (Figure 1).

1. Inclusion criteria for the innovation: there is a spontaneous demand for scaling, the innovation aims to meet a demand for relevant primary care and services in the province of Quebec, the innovation has an epidemiological justification, the innovation is part of a health organization in Quebec, and the innovation has already been piloted and shown to be effective.
2. Inclusion criteria for the innovation team: it has a committee or governance for the development and scaling of the innovation, it is part of a health organization in Quebec, it accepts that the research team participate as an observer in its strategic meetings, it agrees to nominate a person to be the coordinator responsible for the monthly and weekly follow-up with the members of the research team, the innovation team coordinator accepts the responsibility for connecting the research team with managers and health professionals in the target scaling setting, and the innovation team will be available during the research period (1 year) for monthly update meetings to respond to weekly ad hoc email requests from the research team on the innovation and the scaling process.
3. Inclusion criteria for the target site: a specific geographical place is targeted to receive the innovation or there are professionals already using the innovation in the place where it is exposed who are not members of the innovation team, the plan for receiving the scaled innovation is embedded in a health organization in Quebec, an organizational structure is available for receiving the scaled innovation, and partners in the target site are in place to support the scaling plan.
4. Inclusion criteria for individual participants: participants must be at least 18 years old; able to provide informed consent; able to actively participate, read, understand, and communicate in English or French; involved; in contact with or affected by the scaling initiative; and a decision maker in a health and social care organization, a health care provider, or a scaling expert working in the province of Quebec.

#### Support for Scaling

The steering committee will establish a support network for the innovation team. Supporting organizations will be instructed to offer support and advice for scaling without providing a ready-made scaling plan. We will conduct semistructured interviews and focus groups, with the following four categories of interviewees: (1) decision makers from health and social care organizations (n=1-4), (2) health care providers (n=3-8), (3) members of the innovation team responsible for scaling (n=2), and (4) end users (n=2-4).

### Recruitment

The innovation team will be asked to appoint a coordinator who will act as the primary point of contact between the research team and the innovation team. This individual will serve as a facilitator and work closely with decision makers, health care providers, end users, and the research team to ensure effective communication and collaboration. Additionally, the coordinator will be responsible for identifying potential participants and providing their names to the research team for consideration for semistructured interviews and focus groups. Once potential participants have been identified, the research team will send them an email inviting them to participate in the study. The email will outline the main objectives of the research and extend an invitation to participate.

### Qualitative Data Collection

#### Innovation Team

We plan to ask the team about (1) the components of the innovation (including technical requirements and costs) and its impacts on patients and health care professionals, (2) their opinions on how and why it should be scaled, (3) their scaling plan and strategies, and (4) their experiences of creating and implementing the scaling plan. We will also document any challenges encountered and how they were addressed in practice. We will follow developments with weekly emails and monthly follow-up meetings for 1 year.

#### Target Site

We intend to seek input from a range of decision makers (n=1-4), health care professionals (n=3-8), and end users (n=2-4) at the target site, as identified by the innovation team, regarding their firsthand experience with implementing the scaled innovation. We will conduct 1-hour semistructured interviews and focus groups (n=6-8). Participants in both interviews and focus groups will be asked about the innovation and their experience of scaling the health innovation, their view on the successful adaptation or not of the innovation, suggestions for improvement, potential factors influencing its implementation in their context, and possible complementary actions to be put into practice. One-hour individual structured interviews will be conducted face-to-face or on digital platforms (Teams [Microsoft Corp] or Zoom [Zoom Technologies Inc]) and adapted to accommodate the availability of participants. We will hold focus groups with the health care providers lasting 2 hours (maximum) outside office hours to reduce the impact on the services offered. Interviews will be conducted in English or French. One of the research team members with expertise in scaling and training in health care research innovation will conduct the interviews and focus groups. The interviews and focus groups will be recorded and transcribed, while an assistant moderator will be responsible for taking meeting notes during the interviews and focus group sessions. We will also participate as nonparticipant observers in some key meetings of the innovation team or training sessions given by the innovation team. During the observation periods, the research team will take observation notes about the meeting and the scaling process.

### Interview Guide

The interview guide will be based on the 4 McLean and Gargani guiding principles of scaling and will be adapted to the different roles of the interested parties (1 interview guide for decision makers, health professionals, and the innovation team and another one for end users).

We plan to carry out supplementary descriptive quantitative data collection that includes self-administered questionnaires to collect sociodemographic information from each participant (eg, age, sex, gender, civil status, and employment status) involved in the semistructured interviews in addition to a descriptive data collection of organizational characteristics of the target scaling site. The second source of quantitative data will be the various documents describing the scaling process (eg, archival records, reports on the innovation development, minutes of meetings, email exchanges, data from the monitoring of scaling, training manuals and materials, and postintervention feedback) to complement and support our qualitative analysis [34]. We will proceed to content analysis and direct analysis based on the 4 McLean and Gargani guiding principles, providing a comprehensive account of the execution of the scaling plan for detailed analysis.

### Data Analysis

#### Theoretical Model

Using a framework approach [35], we will develop an a priori template of codes based on the McLean and Gargani guiding principles. We will use the data collected, both qualitative (interview transcripts and documentation) and quantitative (questionnaires and documentation), to (1) compare the innovation team’s scaling plan with how scaling was implemented in practice at the target site and (2) determine the concordances or discordances between the 4 principles and participants’ observations regarding their experience of developing the innovation, planning scaling, implementing scaling, and receiving the scaled innovation at the target site.

We will conduct a directed analysis by reading the full transcripts of the focus groups and the semistructured interviews as well as the third-party observations to obtain a sense of the overall data and compare coders’ findings to reach agreement about the key themes identified based on the McLean and Gargani guiding principles. Using intracode methods, the research team will compare findings to reach a consensus on the key themes. Participants will be sent summaries of the results of the interviews and focus groups that they participated in and asked to verify their accuracy. A descriptive analysis of organizational (sites, decision makers, and health care providers) and sociodemographic (decision makers and health care providers) characteristics will also be conducted.

#### Validation

To ensure the internal validity of the analysis, the principal investigators will perform triangulation of data, cross-referencing data from multiple sources. To ensure external validity, a detailed description of the context in which scaling takes place will be provided, that is, the target clinic and its professional health care provider team. To further ensure the accuracy and credibility of the final report, it will be circulated among the collaborators and the committee to validate its content.

### Knowledge Mobilization

Our knowledge mobilization plan is to involve knowledge users (eg, investigators, health care providers, and decision makers) and end users (eg, patient partners) in the steering committee. They will meet every 3 months via teleconference to discuss research progress and provide guidance on research directions. Our team already has extensive experience in the involvement of end users in the design, development, and implementation of health care solutions. Our approach considers the opinions of all end users as equally important. We will send out a newsletter every 4 months for all stakeholders.

We will produce and deliver an adapted and improved scaling plan, based on our results as seen through the lens of the McLean and Gargani guiding principles, to the innovation team. An executive report will be made available free of charge on the research team’s website. We will prepare summaries of our research results tailored to specific knowledge user groups such as clinicians’ organizations (family physicians, nurses, psychologists, and social workers) and researchers (peer-review publications).

Finally, we will disseminate study results in the form of (1) recommendations for preparing and monitoring scaling in primary care with an ethical lens, (2) presentations at scientific and professional conferences, (3) publications in peer-reviewed journals, and (4) through relevant networks such as the Research on Patient-Oriented Scaling-up network, the Strategy for Patient-Oriented Research (SPOR) Evidence Alliance, the Quebec College of Family Physicians and Réseau-1 Québec.

### Ethical Considerations

Ethical approval was obtained from the CIUSSS de la Capitale-Nationale ethics board (MP-13-2023-2770). All stages of this research project will be carried out in accordance with Canadian procedures for informed consent (Tri-Council Policy Statement: Ethical Conduct for Research Involving Humans) [36].

## Results

This study was funded in March 2020 by the Canadian Institutes of Health Research. The steering committee has been formed and is giving expert advice and recommendations. The innovation has been identified, and the innovation team has agreed to contribute to this study by informing potential participants for interviews. Participants recruitment started in November 2023, and data collection began in December 2023. The interview guide has been developed through collaboration with 1 of the authors of the McLean and Gargani guiding principles, and a second interview guide has been adapted for conducting interviews with end users. Results are expected to be published in the first quarter of 2024.

## Discussion

This study aims to use a single descriptive case study to observe, document, and analyze how, in real-life conditions, a promising health care innovation is being or will be spontaneously scaled in a health and social care organization across the province of Quebec. During the process of scaling, many external factors that may not be accounted for in most guidelines, such as the type of innovation being scaled, may influence the outcome. By taking a holistic approach that considers multiple interested parties (eg, patient or citizen partners, decision makers, professionals, researchers, and community members), we will gain a broad perspective on the spontaneous scaling process, enabling us to better understand real-life situations and the challenges that arise during scaling. One of the expected results is a better understanding of the role of flexibility and adaptability in scaling. Few studies have documented the actual process of spontaneous scaling and its practical implications.

By using the McLean and Gargani guiding principles, we also expect our results will give us insight into the ethical aspects of scaling that are overshadowed by the technical aspects and often given insufficient attention, despite their importance. The choice to use this framework was influenced by a need to better define and distinguish ethical considerations in scaling guidelines [5]. While “justification” may seem to be the most critical ethical principle of scaling among the 4, the other 3 also integrate ethical considerations. For instance, “coordination” is sometimes described by McLean and Gargani [9] as *inclusive coordination* because it involves engaging with stakeholders who are usually excluded in impact studies (eg, patients) to ensure the success of the innovation. By using these principles, we can single out the equity-related processes of an innovation and explore its ethical aspects in greater detail.

This study will provide valuable insights into the strategies, challenges, and outcomes of scaling innovations in real-life practical settings. It will provide evidence-based material to support scaling projects to integrate ethical considerations and to incorporate the flexibility to accommodate real-life demands.

## Appendix

Multimedia Appendix 1 Peer review report by the Canadian Institutes of Health Research (CIHR).

## Abbreviations

CIUSSS: Centre Intégré Universitaire de Santé et de Services Sociaux (Integrated University Health and Social Services Center)
CISSS: Centre Intégré de Santé et de Services Sociaux (Integrated Health and Social Services Center)
COREQ: Consolidated Criteria for Reporting Qualitative Research
SPOR: Strategy for Patient-Oriented Research

## Data Availability

The data sets generated and analyzed during this study are available from the corresponding author on reasonable request.
