# Supplementary material for: Spontaneous Scaling of a Primary Care Innovation in Real-Life Conditions: Protocol for a Case Study
Source: JMIR Res Protoc. 2023 Dec 28;12:e54855. doi: 10.2196/54855 (PMC10784976; doi:10.2196/54855)
Supplement: Multimedia Appendix 1 [file resprot_v12i1e54855_app1.pdf]

Review Type/Type d'évaluation: Committee Member 1/Membre de comité 1
Name of Applicant/Nom du chercheur: Légaré, France
Application No./Numéro de demande: 431716
Agency/Agence: CIHR/IRSC
Competition/Concours: 2019-10-15 Operating Grant: Spread and Scale of Existing Community-Based Primary Healthcare and eHealth Innovations/Subv. de fonct.: Diffusion et mise à l'échelle d'innovations existantes en soins de santé communautaires de première ligne et en cybersanté
Committee/Comité: Spread and Scale of Existing CBPHC and eHealth Innovations/Diffusion et mise à l'échelle d'innovations existantes en SSCPL et en cybersanté
Title/Titre: évaluatioN de l'implAntation d'une interVention Interprofessionnelle de la prise de décision partaGée sur la locAlisaTion de sOins avec les aîNés (NAVIGATION)

Assessment/Évaluation:

Dr. Légaré and colleagues propose to expand the use of a tested inter professional approach to shared decision-making that is designed to help older adults and their caregivers make choices about staying at home or moving to another setting in a way that reflects their values and preferences as well as current evidence. It includes an online tutorial, an interactive workshop, a video, and a decision guide. The intent is to spread this approach first in a pilot and then throughout the CIUSSS de la Capitale-Nationale.

Taking advantage of the 'natural experiment' represented by the CIUSSS's intent to spread this approach across the region, the research team intends to undertake focus groups and/or interviews with health professionals, team managers, older adults and their family members. A document analysis is also planned. Specific research objectives include:

(1) Documenting the implementation of the plan to spread this approach across CIUSSS-CN (with 8 sub-territories identified), guided by the McLean and Gargani conceptual framework.

(2) Identifying similarities and differences between actual actions and those suggested by the framework

(3) Producing a guide for spreading a health and social services innovation in primary care in an industrialized country

(4) Launching the basis for an international research group in this domain.

The proposal incorporates a thoughtful approach to GBA+ considerations.

The team includes well-qualified investigators from both Canada and Australia. Members have appropriate interprofessional expertise, as well as expertise derived through lived experience. Relationships have already been established with relevant individuals within CIUSSS-CN, and the application includes participation and a strong support letter from the organization.

The team includes a balance of both experienced and new investigators. While their broad range of expertise is clearly described, the intended roles of individual team members are not clear from the proposal. It appears that the intent is for much of the proposed work to be undertaken by project staff. This includes data collection and analysis. Biographical information for these individuals was not included in the proposal and so their qualifications to undertake the range of responsibilities proposed cannot be evaluated from the information provided, although the team includes individuals who would be in a good position to provide effective supervision for these roles. Likewise, plans for executing objectives 3 and 4 are not clear from the

**Review Type/Type d'évaluation:**	Committee Member 1/Membre de comité 1
**Name of Applicant/Nom du chercheur:**	Légaré, France
**Application No./Numéro de demande:**	431716
**Agency/Agence:**	CIHR/IRSC
**Competition/Concours:**	2019-10-15 Operating Grant: Spread and Scale of Existing Community-Based Primary Healthcare and eHealth Innovations/Subv. de fonct.: Diffusion et mise à l'échelle d'innovations existantes en soins de santé communautaires de première ligne et en cybersanté
**Committee/Comité:**	Spread and Scale of Existing CBPHC and eHealth Innovations/Diffusion et mise à l'échelle d'innovations existantes en SSCPL et en cybersanté
**Title/Titre:**	évaluatioN de l'implAntation d'une interVention Interprofessionnelle de la prise de décision partaGée sur la locAlisaTion de sOins avec les aîNés (NAVIGATION)

Assessment/Évaluation:

proposal.

Note: the included systematic review reflects literature that was tagged with terms such as "scaling up" and "scalability." Significant literature in this field is not, however, tagged with these specific terms. You may wish to consult related literature domains (e.g. change management) that reflect a broader range of interventions and conceptual frameworks than those captured in the systematic review included with this package. Canadian repositories of academic and grey literature of this type are available.

Review Type/Type d'évaluation: Committee Member 2/Membre de comité 2
Name of Applicant/Nom du chercheur: Légaré, France
Application No./Numéro de demande: 431716
Agency/Agence: CIHR/IRSC
Competition/Concours: 2019-10-15 Operating Grant: Spread and Scale of Existing Community-Based Primary Healthcare and eHealth Innovations/Subv. de fonct.: Diffusion et mise à l'échelle d'innovations existantes en soins de santé communautaires de première ligne et en cybersanté
Committee/Comité: Spread and Scale of Existing CBPHC and eHealth Innovations/Diffusion et mise à l'échelle d'innovations existantes en SSCPL et en cybersanté
Title/Titre: évaluatioN de l'implAntation d'une interVention Interprofessionnelle de la prise de décision partaGée sur la locAlisaTion de sOins avec les aîNés (NAVIGATION)

Assessment/Évaluation:

Summary
The team propose to study the large scale implementation of interprofessional approach to shared decision making in home care teams of CIUSSS-Capitale Nationale as a natural experiment to document and develop a guide for large scale primary care implementations. Their objectives are 1) to document the scaling of this approach in home care teams; 2) to compare actions done to those recommended by McLean & Gargani conceptual framework; 3) to produce a primary care innovation scaling guide; and 4) to develop an international research team on scaling. Data collection will include interviews, focus group discussions and analyses of written documents of the scaling process.

Strengths
· The research team already demonstrated the feasibility and benefits of using the interprofessional approach to shared decision making in home care teams in Quebec. They propose to study its large scale implementation in the new political context of Quebec health care system.
· The implementation of interprofessional approach to shared decision making in home care teams is already occurring in CIUSSS-Capitale Nationale. The momentum is thus optimal to study the process of scaling, and learn from it.
· The principal applicant and the team has extensive expertise in studying shared decision making, and leaves no doubt on their capacity to complete this project.
The scaling is occurring naturally, which offers a great momentum to study the process of scaling.

Weaknesses
· The proposal is heavy to read due to the multiple abbreviations used throughout the text.
· The section on potential challenges and mitigation strategies is very thin and focused on the possibility of delays in the naturally occurring scaling. Any risk of challenges in the collaboration between the clinical and research teams? Any potential risk of disagreement between the leaders of the scaling and the leaders of the scaling research?

Budget
The proposed budget seems appropriate.

Review Type/Type d'évaluation: SO Notes /Notes de l'agent scientifique
Name of Applicant/Nom du chercheur: Légaré, France
Application No./Numéro de demande: 431716
Agency/Agence: CIHR/IRSC
Competition/Concours: 2019-10-15 Operating Grant: Spread and Scale of Existing Community-Based Primary Healthcare and eHealth Innovations/Subv. de fonct.: Diffusion et mise à l’échelle d’innovations existantes en soins de santé communautaires de première ligne et en cybersanté
Committee/Comité: Spread and Scale of Existing CBPHC and eHealth Innovations/Diffusion et mise à l’échelle d’innovations existantes en SSCPL et en cybersanté
Title/Titre: évaluatioN de l'implAntation d'une interVention Interprofessionnelle de la prise de décision partaGée sur la locAlisaTion de sOins avec les aîNés (NAVIGATION)

Assessment/Évaluation:

Strengths:

The applicants are a very strong international research team with a range of appropriate expertise and lived experience. The feasibility of the intervention being scaled-up is already well defined. There is strong momentum behind the scale-up and the applicants are an embedded team within a pragmatic implementation, appropriately taking advantage of this context. Relationships are already established with decision makers in the CIUSS, which is participating and has given strong support.

The approach to GBA+ was strong.

Weaknesses:

Four objectives were proposed, but the research approach was described only for the first two.

Scale-up of a mature intervention is already occurring and there was limited description of the actual implementation. Risk mitigation did not consider challenges to researchers engaging with clinical staff. Although there was a letter of support from the managers at the CIUSS, there was no clear indication that the clinical teams where the scale-up will occur are engaged.

The approach proposed is based on a literature review that was conducted with a specific query, which may have missed relevant evidence.

Specific roles of team members were not well defined, and some key aspects of the research will be done by staff to be hired, so it was difficult to access their capacity to conduct the work.

**Review Type/Type d'évaluation:**	SO Notes /Notes de l'agent scientifique
**Name of Applicant/Nom du chercheur:**	Légaré, France
**Application No./Numéro de demande:**	431716
**Agency/Agence:**	CIHR/IRSC
**Competition/Concours:**	2019-10-15 Operating Grant: Spread and Scale of Existing Community-Based Primary Healthcare and eHealth Innovations/Subv. de fonct.: Diffusion et mise à l’échelle d’innovations existantes en soins de santé communautaires de première ligne et en cybersanté
**Committee/Comité:**	Spread and Scale of Existing CBPHC and eHealth Innovations/Diffusion et mise à l’échelle d’innovations existantes en SSCPL et en cybersanté
**Title/Titre:**	évaluatioN de l'implAntation d'une interVention Interprofessionnelle de la prise de décision partaGée sur la locAlisaTion de sOins avec les aîNés (NAVIGATION)

Assessment/Évaluation:

Budget:

No concerns were noted.
